# Neural synchrony examined with magnetoencephalography (MEG) during eye gaze processing in autism spectrum disorders: preliminary findings

**DOI:** 10.1186/1866-1955-6-15

**Published:** 2014-06-17

**Authors:** Renée Lajiness-O’Neill, Annette E Richard, John E Moran, Amy Olszewski, Lesley Pawluk, Daniel Jacobson, Alfred Mansour, Kelly Vogt, Laszlo A Erdodi, Aimee M Moore, Susan M Bowyer

**Affiliations:** 1 Eastern Michigan University, Ypsilanti, MI, USA; 2 Department of Psychiatry, Neuropsychology Section, University of Michigan Health Systems, Ann Arbor, MI, USA; 3 Henry Ford Hospital, Detroit, MI, USA; 4 Department of Psychiatry, SUNY Upstate Medical University, Syracuse, NY, USA; 5 United States Air Force, USMS, Biloxi, MS, USA; 6 Dartmouth, Department of Psychiatry, Geisel School of Medicine at Dartmouth, Lebanon, NH, USA; 7 Wayne State University, Detroit, MI, USA; 8 Oakland University, Rochester, MI, USA

**Keywords:** Autism spectrum disorder, Eye gaze, Neural synchrony, Coherence, Magnetoencephalography, Social cognition

## Abstract

**Background:**

Gaze processing deficits are a seminal, early, and enduring behavioral deficit in autism spectrum disorder (ASD); however, a comprehensive characterization of the neural processes mediating abnormal gaze processing in ASD has yet to be conducted.

**Methods:**

This study investigated whole-brain patterns of neural synchrony during passive viewing of direct and averted eye gaze in ASD adolescents and young adults (*M*_*Age*_ = 16.6) compared to neurotypicals (NT) (*M*_*Age*_ = 17.5) while undergoing magnetoencephalography. Coherence between each pair of 54 brain regions within each of three frequency bands (low frequency (0 to 15 Hz), beta (15 to 30 Hz), and low gamma (30 to 45 Hz)) was calculated.

**Results:**

Significantly higher coherence and synchronization in posterior brain regions (temporo-parietal-occipital) across all frequencies was evident in ASD, particularly within the low 0 to 15 Hz frequency range. Higher coherence in fronto-temporo-parietal regions was noted in NT. A significantly higher number of low frequency cross-hemispheric synchronous connections and a near absence of right intra-hemispheric coherence in the beta frequency band were noted in ASD. Significantly higher low frequency coherent activity in bilateral temporo-parieto-occipital cortical regions and higher gamma band coherence in right temporo-parieto-occipital brain regions during averted gaze was related to more severe symptomology as reported on the Autism Diagnostic Interview-Revised (ADI-R).

**Conclusions:**

The preliminary results suggest a pattern of aberrant connectivity that includes higher low frequency synchronization in posterior cortical regions, lack of long-range right hemispheric beta and gamma coherence, and decreased coherence in fronto-temporo-parietal regions necessary for orienting to shifts in eye gaze in ASD; a critical behavior essential for social communication.

## Background

Orienting to eye gaze is a vital skill present from birth and underlies effective non-verbal communication and social interaction [1] Failure to detect and/or respond in a typical manner to information conveyed by eye gaze is possibly the most important early hallmark of autism spectrum disorders (ASD) [2]. During social interaction, a person’s eyes convey information about their direction of attention, emotion, and mental state [3]. Early responsiveness to eye gaze cues develops quickly and evolves into joint attention, a complex form of non-verbal communication that occurs when one individual follows another individual’s eye gaze or gesture to an object or third individual [1]. Joint attention is essential to the development of both social and language functioning [4-7], yet this pivotal skill fails to develop appropriately in infants with ASD [8,9]. Although research suggests that reflexive responses to eye gaze cues may be intact in individuals with ASD [10,11], studies indicate that orienting voluntarily to eye gaze is impaired and those with ASD may not demonstrate preferential sensitivity to eye gaze as a social cue [12-15]. Instead, it appears that for these individuals, others’ eyes may merely serve as a spatial cue, much like a directional arrow [16].

Altered event-related potentials in response to shifts in eye gaze in 6- to 10-month-olds have been found to be associated with confirmed ASD diagnosis at 36 months of age, suggesting that abnormal response to eye gaze constitutes an important social cognitive endophenotype of ASD [17]. More specifically, in a recent investigation exploring neural responses to eye gaze cues in infants at risk for ASD, Elsabbagh and colleagues [17] reported that typically developing infants and infants at risk for but not diagnosed with ASD at 36 months of age demonstrated a higher P400 amplitude for gaze shifts away *versus* toward the infant, while those with a confirmed diagnosis at 36 months of age did not differentiate the conditions. The failure to appropriately orient to eye gaze in ASD likely has cascading effects on a wide range of developmental tasks including social and communicative functions [18]. However, while brain activation during tasks requiring eye gaze processing has been consistently found to be abnormal in ASD [19-23], the literature to date has not yet provided a coherent understanding of the neural processes underlying abnormal eye gaze processing in ASD.

This study investigated whole-brain patterns of coherence during viewing of direct and averted eye gaze in ASD. Coherence, a measure of synchronization between active cortical network sources within a given frequency band [24], is used to measure short- and long-range connectivity. Theoretical models of ASD such as those proposed by Brock and colleagues [25,26] and Belmonte *et al.*[27] propose that abnormally elevated levels of high-frequency neural activity and over-connectivity within localized brain regions causes impaired discrimination of signal from noise. This in turn causes impaired connectivity between distal cortical regions, leading to reduced activation of cortical regions involved in higher order processing compared to neurotypicals (NT). Recent research has supported these models, confirming that individuals with ASD consistently show abnormal patterns of connectivity between brain regions, with the most consistent finding being a lower degree of connectivity between frontal and posterior regions compared to NT, both at rest and while performing cognitive tasks [28].

Recent reports suggest that disruptions in synchronous neural oscillatory activity are a primary cellular mechanism of impairment in ASD [29], particularly in the gamma frequency band, as identified by resting-state EEG and magnetoencephalography (MEG) [15-17]. Gamma band synchrony has been found to be associated with perceptual binding at early levels of sensory processing, attention [30], and working and long-term memory [31], and is purported to be involved in top-down modulation of sensory signals and large-scale integration of distributed neural networks [32].

Despite recent interest in neural connectivity in ASD, very few MEG studies have examined functional connectivity during face processing, and no studies have examined connectivity during eye gaze processing. In a recent MEG investigation in children and adults with ASD, the gamma frequency band response in right lateral occipital areas was largely absent compared to typically developing participants while viewing emotions on faces [33]. Khan and colleagues also described abnormalities in gamma oscillatory activity during face processing in ASD compared to typically developing adolescents and young adults [33]. The authors focused on nesting oscillations in which the amplitude or phase of a lower frequency band (for example, alpha) modulates the phase or amplitude of a higher frequency band, referred to as phase amplitude coupling (PAC). Group differences in alpha-gamma PAC were noted but in the absence of reductions in alpha or gamma power, suggesting these differences were driven by variation in the timing of gamma generation. In addition, statistically significant differences in coherence in the alpha band in the inferior frontal gyrus, anterior cingulate, and left precuneus were noted, consistent with reductions in long-range connectivity. Reductions in local functional connectivity within the fusiform face area (FFA) were also reported.

The central role of eye gaze processing impairment in ASD makes eye gaze a good candidate for elucidating aberrant patterns of connectivity and as a potential biomarker. We utilized MEG to record patterns of neural activation during direct and averted eye gaze processing in ASD and NT, enabling calculation of coherence between circumscribed cortical regions within discrete frequency bands. Specifically, we calculated coherence within the beta and gamma frequency bands, which are currently implicated in long- and short-range transmission of information involved in high-level cognitive processing, respectively [34]. As an exploratory aim, we also examined a ‘lower’ frequency band, collapsing delta, theta, and alpha bands for comparison with the alert working brain, beta, and gamma frequencies.

## Methods

### Participants

Eighteen participants completed the study; 10 participants with ASD (*M*_Age_ = 16.6); *M*_IQ_ = 112) and eight neurotypical controls (*M*_Age_ = 17; *M*_IQ_ = 116). The groups did not differ significantly in age (*U*(16) = 1.79, *P* = 0.76), gender (*χ*^2^ = 0.11), or Full Scale IQ, with both groups generally performing in the Average to Above Average range on the Wechsler Abbreviated Scale of Intelligence (WASI; *U*(16) = 40, *P* = 0.74) [35]. See Table 1 for a review of the demographic variables. There was equal age distribution between the genders of the two groups. To ensure that potential developmental differences between the groups did not account for our effect, significant relationships between our brain region coherence values with age were inspected. Age was related to coherence values in the beta frequency band only in the right inferior frontal to right superior temporal pathway in the NT controls (*r* = 0.52, *P* = 0.02). Five of the 10 ASD participants were on psychotropic medications. Two participants were prescribed a single medication, an antidepressant (Prozac) or antipsychotic (Risperdal). Two participants were prescribed an antidepressant and psychostimulant (Prozac or Zoloft and Concerta), and one participant was prescribed three medications, an antidepressant, anxioltyic, and mood stabilizer (Cymbalta, Buspar, and Depakote).

**Table 1:** Demographic characteristics of the participants

**ASD subjects**	**Gender**	**Age**	**Dominance**	**FSIQ standard score**	**Vocabulary T-score**	**Matrix reasoning T-score**	**ADI-R social**	**ADI-R communication**	**ADIR repetitive behaviors**
1	M	19.00	R	131.0	77.00	57.00	14.00	16.00	9.00
2	F	13.00	L	96.0	35.00	61.00	23.00	15.00	4.00
3	M	13.00	R	93.0	39.00	53.00	27.00	24.00	6.00
4	M	15.00	R	137.0	67.00	73.00	20.00	14.00	8.00
5	M	19.00	R	144.0	77.00	67.00	17.00	9.00	3.00
6	F	16.00	R	119.0	62.00	60.00	20.00	11.00	5.00
7	M	24.00	R		Discontinued	66.00	24.00	18.00	3.00
8	F	16.00	R	79.0	44.00	28.00	20.00	18.00	6.00
9	M	16.00	R	118.0	61.00	60.00	13.00	10.00	7.00
10	M	15.00	R	92.0	64.00	28.00	17.00	16.00	8.00
*Mean*		16.60		112.1	58.44	55.30	19.50	15.10	5.90
*SD*		3.31		22.9	15.61	15.42	4.40	4.46	2.13
*Range*		11-24		79-144	35-77	28-73			
**Control subjects**									
1	M	21.00	R	122.0	64.00	61.00			
2	F	13.00	R	120.0	66.00	57.00			
3	M	13.00	R	122.0	59.00	66.00			
4	M	15.00	R	118.0	60.00	60.00			
5	M	19.00	R	117.0	58.00	60.00			
6	F	19.00	L	119.0	59.00	63.00			
7	M	18.00	R	100.0	44.00	57.00			
8	F	18.00	R	117.0	60.00	57.00			
*Mean*		17.00		116.9	58.75	60.13			
*SD*		2.98		7.1	6.56	3.23			
*Range*		13-21		100-122	44-66	57-66			
*Mann–Whitney U P value*	0.11	0.76		0.70	0.54	0.90			

ADI-R = Autism Diagnostic Interview-Revised; ASD = autism spectrum disorder; FSIQ = Full Scale Intelligence Quotient.

Individuals were recruited from and underwent MEG procedures at Henry Ford Hospital (HFH). Participants were diagnosed with Pervasive Developmental Disorder (PDD) (recently revised to ASD) based on the Diagnostic and Statistical Manual of Mental Disorders-Fourth Edition-Text Revision (DSM-IV-TR) [2] diagnostic criteria. Diagnoses were confirmed with the Autism Diagnostic Interview-Revised (ADI-R) [36]. Means and standard deviations for the ADI-R domain scores include: *M*_Social_ (*SD*) = 19.25 (4.59); *M*_Communication_ (*SD*) = 15.50 (4.34); *M*_Repetitive_ (*SD*) = 6.63 (1.69). Inclusion criteria included at least low average intelligence (≥80 Full Scale IQ scores on the WASI). Exclusionary criteria included any known history of head injury with unconsciousness, epilepsy, affective or anxiety disorders. No ASD participant had a history of a genetic disorder. NT participants had no history of developmental delay, learning disorder, or ASD in a first-degree relative. All APA Ethical Guidelines were followed and Institutional Review Board approval was obtained from all institutions participating in this study.

### MEG data acquisition and preprocessing

Cortical activity was recorded using a 148 channel whole head MEG system (4D Neuroimaging, Magnes WH2500) with magnetometer type sensors. During acquisition, the data were band-pass filtered 0.1 to 100 Hz and digitally sampled at 508.63 Hz. The timing of stimuli was recorded as pulse codes (representing the type of stimulus) on a trigger channel simultaneously collected with the MEG data. In postprocessing, noise artifacts due to heart and body movement were eliminated using an independent component analysis (ICA). Singular valued decomposition was used to remove any other artifacts in the data such as mouth movements if needed. Regarding movement artifact, runs are repeated if the coil on head positions exceeds 0.5 cm, although this did not occur during acquisition. As such, we combined the runs before source reconstruction. Regarding noise reduction, 4D Neuroimaging incorporates a set of reference sensors that are used to sample the environmental magnetic fields and create a file that has a set of weights. These weights are subtracted from the data during data collection. Data were band-pass filtered from 1 to 50 Hz. The locations of events on the trigger and response channels were used to select 2-s epochs of MEG data to examine average evoked responses for the stimuli requiring a conditional button press (that is, asterisk, face, or words). All trials within each condition were averaged to determine the evoked response. All epochs had a baseline of 500 ms before stimuli onset and 1,500 ms of data after stimuli onset.

### Gaze cueing paradigm

**Figure 1:**
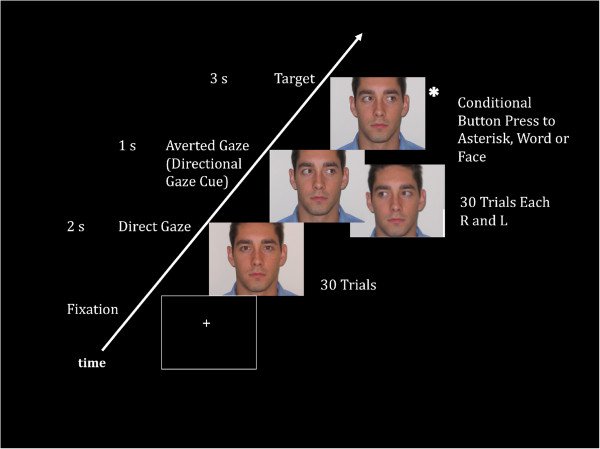
**Gaze paradigm.** Analyses included passive conditions corresponding to slides with direct and averted gaze. In this example, the target slide would require a button press as the character’s gaze is looking at the asterisk. This was how attention to eye gaze was ensured.

The gaze cueing paradigm used was an adaptation of a paradigm used by Pelphrey and colleagues [37]. MEG field responses to gaze cues were collected for two 14-min trials in which five task conditions were administered: direct gaze, averted gaze, and gaze cueing to peripheral stimuli (asterisk, word, or face). In the direct and averted gaze conditions, participants passively viewed a central character. There were 30 trials in the direct gaze condition and 30 each (left and right) in the averted gaze condition. In each of the three additional gaze cueing conditions, the central character engaged in a random gaze shift toward the right or left for 1 s. A target (asterisk, word, or face) then appeared at either the right or the left of the subject for 3 s. See Figure 1 for an example of one of the target stimuli. The next trial began with the character returning to a direct gaze for 2 s with no stimuli in the periphery. The location of the target stimulus was either congruent or incongruent with the direction of the character’s gaze. Sixty targets were presented in each gaze-cueing condition, including 30 congruent and 30 incongruent trials. A conditional button press during gaze cues to the peripheral stimuli conditions ensured engagement during the passive conditions. For the purposes of this analysis, only passive viewing of direct and averted gaze was considered. Results from the evoked response data requiring active responses to the conditional stimuli (asterisk, faces, words) have been previously published [38].

### MEG coherence analysis

Synchronization of neuronal activity was quantified by calculating coherence between cortical sites from MEG imaged brain activations [39,40]. A model of the cortical brain surface was created from an age- and gender-appropriate standard MRI. The MRI was segmented and the brain surface was represented by a cortical model of approximately 4,000 dipoles each having an x, y, and z orientation at each site. Sites were distributed to represent the same volume of cortical gray matter. This model was then morphed to fit the digitized head shape collected during the MEG acquisition. To calculate coherence [40], the MEG data were first divided into 80 segments each containing 7.5-s segments of data and cortical activity in each segment was imaged on to the MRI using the MR-FOCUSS imaging technique [41]. Using the time sequence of imaged activity, coherence between active cortical model sites was calculated for each data segment and then averaged for the completed study. In addition, for each cortical model site, connectivity was quantified by a histogram of the number of sites to which the site had the same level of coherence. Statistical analysis of cortical coherence levels (0 to 1) were used to quantify differences in network connectivity between groups. Changes in coherence and connectivity between brain regions implicated as having deviant electrophysiological activity in the ASD brain were quantified and subjected to further statistical analysis.

**Figure 2:**
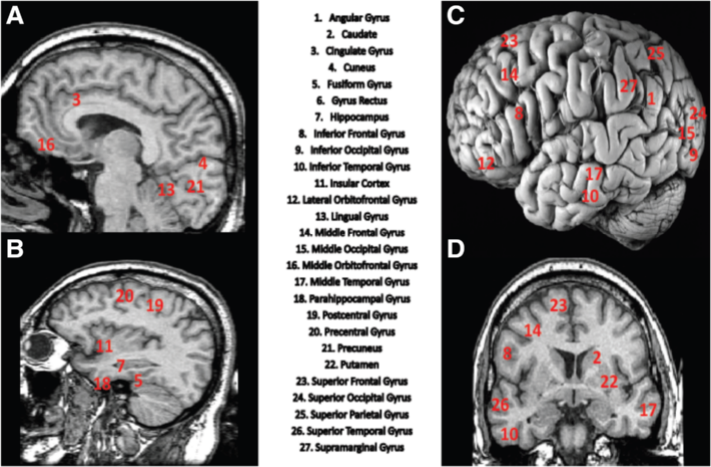
**Brain regions used to develop 1,431 brain region pathways.** Total brain regions equal 54: 27 right and 27 left hemisphere. **(A)** Midsagittal, **(B)** sagittal, **(C)** lateral (postmortem), and **(D)** coronal MR-T1 weighted images revealing 25 of the 27 brain regions. Gyrus rectus and middle orbitofrontal were unable to be visualized given that all x, y, and z locations cannot be viewed concurrently.

Power spectra for activity at all active sites were also calculated and used to quantify differences in low frequency, beta, and gamma power. For the ‘lower’ frequency band, delta, theta, and alpha bands were collapsed for comparison with the alert working brain, specifically beta and gamma frequencies. For additional details of MEG coherence imaging, see our publication [40]. A region-of-interest (ROI) tool implemented in MEG Tools was used to identify 54 regions in the brain (27 in each hemisphere). See Figure 2 for a list of the brain regions. MEG Tools uses a non-linear volumetric transformation of the patient’s brain to transform MEG coordinates to standard Talairach [42] or MNI [43] coordinates [44]. This enables the ROI tool to access an atlas of Brodmann’s area identifiers and an atlas of cortical structures [43].

### Group difference testing

For each frequency band (low, beta, and gamma) within each condition (direct and averted gaze), a *t*-test was conducted to assess group difference in average coherence values for each pair of brain regions (N = 1,431). A *P* value was produced for each region pair. The false discovery rate (FDR) was used to adjust for multiple testing. Because of the large number of tests being performed simultaneously, using a significance level of alpha = 0.05 without adjusting for multiple testing would lead to a large number of false positive results. Bonferroni adjustments for multiple comparisons aim to control the Family Wise Error Rate. If a Bonferroni correction were applied to every test, there would be only a 5% chance of at least one false positive in all of the N = 1,431 tests. Bonferroni corrections require the *P* value to be less than 0.05/N, where N is the number of tests. With N = 1,431, this criterion becomes especially stringent, and many true differences may be missed (false negatives). In the era of large scale testing, a less conservative approach to adjusting for multiple testing has been developed. The FDR is the proportion of tests declared significant that are actually different only due to chance (or the proportion of significant tests that are false positives). The FDR is a widely accepted, less conservative approach to adjusting for multiple testing in large scale problems. The Benjamini-Hochberg algorithm [45] was used to control the FDR at 0.10. From each *t*-test, a *z*-score was computed according to the method of Efron [46] to summarize the difference in coherence values between ASD and NT. Positive *z*-scores indicate higher coherence in the ASD group. A series of chi-squares were computed to determine if the number of intra-hemispheric and inter-hemispheric cortical differences within the low (0 to 15 Hz), beta (15 to 30 Hz), and low gamma (30 to 45 Hz) frequency bands were statistically different between the groups.

To examine relationships between ASD clinical symptoms as reported on the ADI-R and neural oscillatory activity (coherence), a series of Kendall Tau correlation coefficients were computed.

## Results

### Behavioral data

There were no significant between group differences noted in error rates in responding to the conditional button press during the intervening task condition with respect to accuracy (*t*(16) = 0.70 *P* = 0.51), suggesting that both groups were equally engaged in the task. Reaction times were not statistically different (*t*(16) = -0.11 *P* = 0.92). See Table 2 for the accuracy (Total Correct Responses) and reaction time values for correct responses for the NT and ASD groups.

**Table 2:** Average accuracy (total correct responses) and reaction time values for correct responses for each ASD and neurotypical participant

**Total correct responses**		**Reaction time for correct responses**	
**ASD**	**Neurotypical**	**ASD**	**Neurotypical**
47	45	673	915
43	46	1,044	916
46	47	818	887
37	44	1,096	717
45	45	683	718
38	45	936	855
53	45	935	862
45	38	761	929
45		557	
41		1,136	
*M* (*SD*) 44.00 (4.62)	*M* (*SD*) 44.38 (2.72)	*M* (*SD*) 863.90 (196.26)	*M* (*SD*) 849.88 (88.15)

*M* = Mean; *SD* = Standard Deviation.

Values reflect averages over the two runs.

### MEG analysis: coherence imaging of connectivity

#### Direct gaze condition

During the direct gaze condition, 91 of the 1,431 pathways were found to be significantly different between the groups. In NT, higher coherence was observed between frontal, temporal, and parietal regions. Higher coherence was particularly evident between bilateral frontal (middle, inferior, and orbitofrontal) gyri and right superior temporal, pre- and postcentral gyri. In ASD participants, higher coherence was noted between left occipito-parietal (angular, middle, and superior occipital gyri) and bilateral occipito-parietal regions (inferior, middle, superior occipital gyri, and supramarginal regions). See Table 3 for brain region pairs with statistically significant between group differences in coherence for direct gaze collapsed across frequency bands. Only the top 10 regions for each group are presented to simplify the results. No significant group differences were found within the separate frequency bands during the direct gaze condition.

**Table 3:** Brain region pairs with statistically significant between group differences in coherence for direct gaze collapsed across frequency bands

**Brain region pairs (pathway) for direct gaze**	***z*-score**	***P *** **value**
Regions with higher coherence in NT		
R. middle frontal gyrus and R. superior temporal gyrus	-4.69	<0.0001
L. lateral orbitofrontal gyrus and R. precentral gyrus	-4.44	<0.0001
L. lateral orbitofrontal gyrus and R. postcentral gyrus	-4.41	<0.0001
R. inferior frontal gyrus and R. middle frontal gyrus	-4.33	<0.0001
L. superior frontal gyrus and R. superior temporal gyrus	-4.32	<0.0001
L. inferior frontal gyrus and R. postcentral gyrus	-4.26	<0.0001
L. inferior frontal gyrus and R. precentral gyrus	-4.14	<0.0001
R. middle frontal gyrus and R. postcentral gyrus	-4.19	<0.0001
L. middle frontal gyrus and R. precentral gyrus	-4.11	0.0001
L. middle frontal gyrus and R. postcentral gyrus	-4.12	0.0001
Regions with higher coherence in ASD		
L. angular gyrus and L. middle occipital gyrus	5.10	<0.0001
L. angular gyrus and R. middle occipital gyrus	4.77	<0.0001
L. angular gyrus and L. superior occipital gyrus	4.09	<0.0001
L. angular gyrus and R. cuneus	4.02	0.0001
L. angular gyrus and L. inferior occipital gyrus	4.02	0.0001
L. postcentral gyrus and L. superior occipital gyrus	3.86	0.0001
L. angular gyrus and R. inferior occipital gyrus	3.85	0.0001
L. superior occipital gyrus and L. supramarginal gyrus	3.85	0.0001
L. middle occipital gyrus and R. middle occipital gyrus	3.84	0.0001
L. angular gyrus and L. supramarginal gyrus	3.71	0.0002

Only top 10 for each group of the 91 total are reported to simplify results.

#### Averted gaze condition

**Figure 3:**
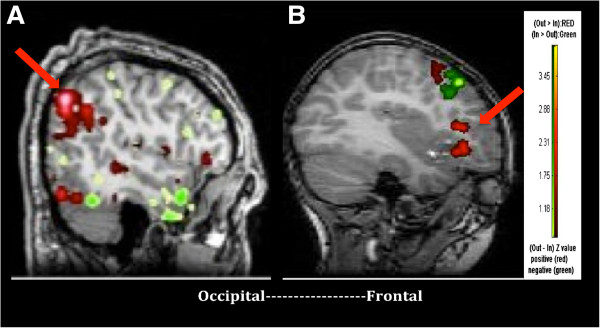
**Subset of regions of network of greatest average coherence in a representative ASD and NT participant.** Red reflects sending of information while green reflects receiving of information flow in areas of high average coherence. In a male adolescent with ASD **(A)**, information in posterior cortical areas (parietal cortex) is highly active in sending information when processing eye gaze cues while minimal frontal activity is noted. In a NT adolescent male **(B)**, the left inferior and middle frontal gyri are highly active in sending information when processing gaze cues.

During the averted gaze condition, 390 of the 1,431 pathways were found to be significantly different between the groups. Consistent with the direct gaze findings, NT demonstrated significantly higher coherent activity across all frequencies in fronto-temporo-parietal regions, consistent with known neuroanatomical substrates critical for responding to shifts in eye gaze (see Figure 3). That is, significantly higher coherence was noted between bilateral frontal (inferior, middle, superior, orbitofrontal gyri) and right frontal (inferior, middle, superior, and precentral gyri), superior temporal, and parietal (postcentral gyrus) regions. ASD participants displayed higher coherence between left parieto-occipital (angular, inferior, and middle occipital) and bilateral temporo-parieto-occipital regions (inferior, middle, superior temporal, occipital, angular gyri) (see Figure 3).

**Figure 4:**
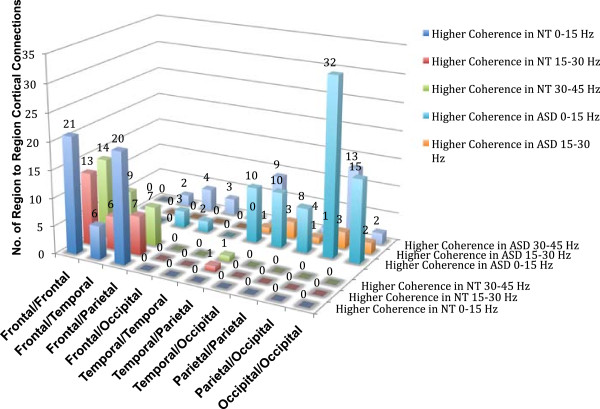
**Regional cortical differences in synchronous beta and gamma activity during averted gaze.** Number of region-to-region cortical differences within the low (0 to 15 Hz), beta (15 to 30 Hz), and low gamma (30 to 45 Hz) frequency bands in regions of either statistically higher coherence in NT or ASD participants.

In contrast to direct gaze, statistically significant between group differences were noted within each frequency during the averted gaze condition. Of the 390 pathways, significant differences in coherence were found within specific frequencies in 233 of the pathways; 127 in the low frequency band, 37 in the beta frequency band, and 69 in the low gamma frequency band. Table 4 presents the brain region pairs with statistically significant between group differences in coherence for averted gaze in the low (0 to 15 Hz), beta (15 to 30 Hz), and gamma (30 to 45 Hz) frequency bands.In the low frequency band, ASD participants displayed higher coherent activity between left parieto-occipital regions and right temporo-parieto-occipital regions and significantly lower coherence between bilateral frontal and right fronto-temporo-parietal regions. In the beta band, ASD participants demonstrated higher coherence between left parieto-occipital regions and bilateral temporo-occipital and left parietal regions. In the gamma band, ASD participants showed higher coherence between bilateral temporo-parieto-occipital regions as well as bilateral parietal and orbitofrontal regions. In both the beta and gamma frequency bands, ASD participants showed lower coherence between bilateral frontal, fronto-temporal, and temporo-parietal regions compared to NT (See Figure 4). In contrast, NT subjects displayed significantly higher coherence between bilateral frontal, fronto-temporal, and fronto-parietal regions across all frequency bands. Due to the essentially non-overlapping distributions, formal chi-square or log linear analyses could not be computed. The number of region-to-region cortical connection counts within the low (0 to 15 Hz), beta (15 to 30 Hz), and low gamma (30 to 45 Hz) frequency bands can be visually inspected in Figure 4.

**Table 4:** Brain region pairs with statistically significant between group differences in coherence for averted gaze in the (A) low (0 to 15 Hz), (B) beta (15 to 30 Hz), and (C) gamma (30 to 45 Hz) frequency bands

**(A) Brain Region Pairs (Pathway) for 0 to 15 Hz**	***z*-score**	***P *** **value**
Regions with higher coherence in NT		
R. inferior frontal gyrus and R. middle frontal gyrus	-5.23	<0.0001
R. inferior frontal gyrus and R. superior frontal gyrus	-4.96	<0.0001
L. lateral orbitofrontal gyrus and R. postcentral gyrus	-4.2	<0.0001
R. superior frontal gyrus and R. superior temporal gyrus	-4.2	<0.0001
R. postcentral gyrus and R. superior frontal gyrus	-4.07	0.00010
L. precentral gyrus and R. superior frontal gyrus	-4.02	0.00020
L. superior frontal gyrus and R. postcentral gyrus	-3.65	0.00030
L. inferior frontal gyrus and R. postcentral gyrus	-3.65	0.00040
L. lateral orbitofrontal gyrus and R. middle frontal gyrus	-3.61	0.00030
L. inferior frontal gyrus and R. inferior frontal gyrus	-3.61	0.00030
L. superior temporal gyrus and R. middle frontal gyrus	-3.55	0.00040
L. precentral gyrus and R. middle frontal gyrus	-3.52	0.00080
R. middle frontal gyrus and R. superior temporal gyrus	-3.51	0.00060
L. lateral orbitofrontal gyrus and R. supramarginal gyrus	-3.49	0.00060
R. middle frontal gyrus and R. postcentral gyrus	-3.49	0.00080
L. middle frontal gyrus and R. postcentral gyrus	-3.42	0.00100
L. lateral orbitofrontal gyrus and R. inferior frontal gyrus	-3.3	0.00130
L. middle frontal gyrus and R. inferior frontal gyrus	-3.27	0.00110
L. middle frontal gyrus and R. precentral gyrus	-3.27	0.00150
R. superior frontal gyrus and R. supramarginal gyrus	-3.26	0.00170
L. lateral orbitofrontal gyrus and R. precentral gyrus	-3.25	0.00130
L. superior temporal gyrus and R. superior frontal gyrus	-3.24	0.00120
L. precentral gyrus and L. superior frontal gyrus	-3.22	0.00190
R. precentral gyrus and R. superior frontal gyrus	-3.2	0.00150
R. middle frontal gyrus and R. superior frontal gyrus	-3.14	0.00170
L. lateral orbitofrontal gyrus and L. precentral gyrus	-3.14	0.00220
L. superior frontal gyrus and R. inferior frontal gyrus	-3.1	0.00200
L. inferior frontal gyrus and R. precentral gyrus	-3.08	0.00250
R. angular gyrus and R. superior frontal gyrus	-3.07	0.00320
L. inferior frontal gyrus and R. middle frontal gyrus	-3.05	0.00230
L. postcentral gyrus and R. superior frontal gyrus	-3.02	0.00320
L. inferior frontal gyrus and R. supramarginal gyrus	-3.02	0.00320
L. superior frontal gyrus and R. precentral gyrus	-3	0.00270
R. middle frontal gyrus and R. supramarginal gyrus	-2.92	0.00470
L. superior parietal gyrus and R. superior frontal gyrus	-2.88	0.00490
L. precuneus and R. superior frontal gyrus	-2.83	0.00730
L. inferior frontal gyrus and L. precentral gyrus	-2.82	0.00560
L. superior frontal gyrus and R. supramarginal gyrus	-2.81	0.00610
L. lateral orbitofrontal gyrus and R. angular gyrus	-2.8	0.00570
L. lateral orbitofrontal gyrus and L. superior parietal gyrus	-2.78	0.00650
L. middle orbitofrontal gyrus and R. postcentral gyrus	-2.78	0.00700
L. middle frontal gyrus and R. supramarginal gyrus	-2.78	0.00720
L. lateral orbitofrontal gyrus and L. precuneus	-2.76	0.00760
L. superior temporal gyrus and R. inferior frontal gyrus	-2.72	0.00660
L. middle frontal gyrus and L. precentral gyrus	-2.71	0.00880
R. middle frontal gyrus and R. precentral gyrus	-2.68	0.00850
L. superior frontal gyrus and R. superior temporal gyrus	-2.66	0.00780
Regions with higher coherence in ASD		
L. angular gyrus and L. inferior occipital gyrus	4.86	<0.0001
L. angular gyrus and R. lingual gyrus	4.79	<0.0001
L. middle occipital gyrus and R. cuneus	4.7	<0.0001
L. middle occipital gyrus and R. lingual gyrus	4.51	<0.0001
L. angular gyrus and L. middle occipital gyrus	4.47	<0.0001
L. angular gyrus and R. middle occipital gyrus	4.46	<0.0001
L. angular gyrus and R. cuneus	4.38	<0.0001
L. inferior occipital gyrus and R. lingual gyrus	4.28	<0.0001
L. angular gyrus and R. superior occipital gyrus	4.17	<0.0001
L. middle occipital gyrus and R. superior occipital gyrus	4.16	<0.0001
L. middle occipital gyrus and R. middle occipital gyrus	4.11	<0.0001
L. inferior occipital gyrus and R. cuneus	4.01	0.00010
L. cuneus and R. lingual gyrus	3.99	0.00010
L. middle occipital gyrus and R. superior parietal gyrus	3.9	0.00010
R. lingual gyrus and R. middle occipital gyrus	3.83	0.00010
L. inferior occipital gyrus and R. superior parietal gyrus	3.74	0.00020
L. angular gyrus and R. inferior occipital gyrus	3.72	0.00020
L. angular gyrus and L. cuneus	3.68	0.00020
R. cuneus and R. middle occipital gyrus	3.6	0.00040
L. middle occipital gyrus and R. inferior occipital gyrus	3.59	0.00040
R. gyrus rectus and R. lingual gyrus	3.57	0.00060
L. angular gyrus and L. lingual gyrus	3.55	0.00040
L. middle temporal gyrus and R. lingual gyrus	3.54	0.00040
L. angular gyrus and L. fusiform gyrus	3.54	0.00060
R. cuneus and R. lingual gyrus	3.51	0.00060
R. middle occipital gyrus and R. superior occipital gyrus	3.47	0.00050
L. middle occipital gyrus and R. angular gyrus	3.47	0.00050
L. middle occipital gyrus and R. middle temporal gyrus	3.47	0.00050
L. superior temporal gyrus and R. cuneus	3.45	0.00070
L. middle temporal gyrus and R. cuneus	3.44	0.00070
L. lingual gyrus and R. lingual gyrus	3.41	0.00070
L. inferior temporal gyrus and R. cuneus	3.39	0.00070
L. inferior occipital gyrus and R. middle occipital gyrus	3.34	0.00090
L. angular gyrus and L. inferior temporal gyrus	3.34	0.00110
R. lingual gyrus and R. superior occipital gyrus	3.32	0.00090
L. inferior occipital gyrus and R. superior occipital gyrus	3.31	0.00090
L. postcentral gyrus and R. cuneus	3.3	0.00100
L. fusiform gyrus and R. cuneus	3.3	0.00110
L. angular gyrus and R. superior parietal gyrus	3.26	0.00120
L. inferior temporal gyrus and R. lingual gyrus	3.25	0.00120
L. inferior occipital gyrus and R. middle temporal gyrus	3.23	0.00140
R. middle occipital gyrus and R. superior parietal gyrus	3.21	0.00130
R. cuneus and R. superior occipital gyrus	3.19	0.00150
L. cuneus and R. middle occipital gyrus	3.19	0.00160
L. angular gyrus and L. middle temporal gyrus	3.17	0.00160
L. superior temporal gyrus and R. lingual gyrus	3.16	0.00170
L. angular gyrus and L. superior occipital gyrus	3.12	0.00190
L. cuneus and R. cuneus	3.1	0.00230
R. cuneus and R. gyrus rectus	3.09	0.00240
L. cuneus and R. inferior occipital gyrus	3.08	0.00210
L. angular gyrus and R. middle temporal gyrus	3.04	0.00250
L. lingual gyrus and R. cuneus	3.02	0.00280
L. cuneus and L. fusiform gyrus	3.01	0.00290
L. inferior occipital gyrus and R. angular gyrus	2.98	0.00300
L. cuneus and L. middle occipital gyrus	2.98	0.00380
L. middle occipital gyrus and R. precuneus	2.95	0.00320
L. postcentral gyrus and R. lingual gyrus	2.94	0.00340
L. inferior occipital gyrus and L. middle occipital gyrus	2.94	0.00480
L. postcentral gyrus and R. superior occipital gyrus	2.88	0.00460
R. superior occipital gyrus and R. superior parietal gyrus	2.87	0.00410
L. inferior occipital gyrus and R. inferior occipital gyrus	2.86	0.00450
L. angular gyrus and R. angular gyrus	2.85	0.00440
L. lingual gyrus and R. middle occipital gyrus	2.83	0.00480
L. fusiform gyrus and R. lingual gyrus	2.83	0.00490
L. middle occipital gyrus and R. lateral orbitofrontal gyrus	2.81	0.00500
L. postcentral gyrus and L. superior occipital gyrus	2.81	0.00530
R. lingual gyrus and R. superior parietal gyrus	2.78	0.00540
L. cuneus and R. superior parietal gyrus	2.78	0.00560
L. middle occipital gyrus and L. middle temporal gyrus	2.76	0.00750
L. middle occipital gyrus and R. supramarginal gyrus	2.75	0.00630
L. precentral gyrus and R. cuneus	2.74	0.00630
R. lingual gyrus and R. middle temporal gyrus	2.74	0.00680
R. inferior occipital gyrus and R. lingual gyrus	2.73	0.00650
L. cuneus and R. superior occipital gyrus	2.7	0.00740
L. angular gyrus and R. gyrus rectus	2.69	0.00740
R. middle occipital gyrus and R. middle temporal gyrus	2.69	0.00740
L. angular gyrus and L. superior temporal gyrus	2.68	0.00740
L. cuneus and L. postcentral gyrus	2.67	0.00770
L. angular gyrus and R. precuneus	2.67	0.00790
L. middle occipital gyrus and R. superior temporal gyrus	2.64	0.00850
**(B) Brain region pairs (pathway) for 15 to 30 Hz**	*z*-score	*P* value
Regions with higher coherence in NT		
R. middle frontal gyrus and R. superior temporal gyrus	-5.18	<0.0001
R. inferior frontal gyrus and R. superior frontal gyrus	-5.18	0.0025
L. lateral orbitofrontal gyrus and R. precentral gyrus	-4.58	<0.0001
R. inferior frontal gyrus and R. superior temporal gyrus	-4.55	<0.0001
L. superior frontal gyrus and R. precentral gyrus	-4.21	<0.0001
R. middle frontal gyrus and R. precentral gyrus	-4.13	<0.0001
R. middle frontal gyrus and R. postcentral gyrus	-4.09	<0.0001
L. superior frontal gyrus and R. superior temporal gyrus	-3.94	<0.0001
L. superior frontal gyrus and R. postcentral gyrus	-3.92	<0.0001
R. precentral gyrus and R. superior temporal gyrus	-3.9	<0.0001
R. superior frontal gyrus and R. superior temporal gyrus	-3.9	0.0001
R. inferior frontal gyrus and R. precentral gyrus	-3.87	0.0001
R. inferior frontal gyrus and R. middle frontal gyrus	-3.84	0.0001
R. precentral gyrus and R. superior frontal gyrus	-3.76	0.0003
R. postcentral gyrus and R. superior frontal gyrus	-3.58	0.0005
L. middle frontal gyrus and R. precentral gyrus	-3.55	0.0005
L. inferior frontal gyrus and R. precentral gyrus	-3.48	0.0007
R. lateral orbitofrontal gyrus and R. precentral gyrus	-3.32	0.0009
L. lateral orbitofrontal gyrus and R. postcentral gyrus	-3.3	0.0001
R. middle orbitofrontal gyrus and R. precentral gyrus	-3.26	0.0017
R. postcentral gyrus and R. superior temporal gyrus	-3.22	0.0013
R. inferior frontal gyrus and R. postcentral gyrus	-3.22	0.0014
L. middle frontal gyrus and R. superior temporal gyrus	-3.17	0.0017
L. middle orbitofrontal gyrus and R. precentral gyrus	-3.16	0.0022
R. middle orbitofrontal gyrus and R. postcentral gyrus	-3.14	0.0024
L. middle frontal gyrus and R. postcentral gyrus	-3.1	0.0021
L. superior frontal gyrus and R. inferior frontal gyrus	-3.07	0.0022
Regions with higher coherence in ASD		
L. angular gyrus and L. middle occipital gyrus	3.66	0.0003
L. superior occipital gyrus and L. supramarginal gyrus	3.65	0.0003
L. middle occipital gyrus and L. middle temporal gyrus	3.58	0.0004
L. angular gyrus and R. middle occipital gyrus	3.51	0.0005
L. middle occipital gyrus and R. middle temporal gyrus	3.48	0.0006
L. superior occipital gyrus and R. inferior occipital gyrus	3.21	0.0014
L. angular gyrus and L. supramarginal gyrus	3.19	0.0014
L. middle occipital gyrus and L. superior occipital gyrus	3.15	0.0017
L. middle occipital gyrus and L. superior temporal gyrus	3.08	0.0022
L. middle temporal gyrus and L. supramarginal gyrus	3.08	0.0022
**(C) Brain region pairs (pathway) for 30 to 45 Hz**	*z*-score	*P* value
Regions with higher coherence in NT		
L. lateral orbitofrontal gyrus and R. precentral gyrus	-4.04	0.0001
L. middle frontal gyrus and R. precentral gyrus	-3.99	0.0001
R. middle frontal gyrus and R. superior temporal gyrus	-3.86	0.0002
L. inferior temporal gyrus and R. precentral gyrus	-3.79	0.0002
L. middle frontal gyrus and R. postcentral gyrus	-3.76	0.0003
R. middle frontal gyrus and R. postcentral gyrus	-3.74	0.0005
R. middle frontal gyrus and R. precentral gyrus	-3.72	0.0003
R. inferior frontal gyrus and R. middle frontal gyrus	-3.64	0.0003
L. middle frontal gyrus and R. superior temporal gyrus	-3.6	0.0003
L. inferior frontal gyrus and R. precentral gyrus	-3.6	0.0004
L. middle orbitofrontal gyrus and R. precentral gyrus	-3.59	0.0004
L. lateral orbitofrontal gyrus and R. postcentral gyrus	-3.53	0.0006
L. superior frontal gyrus and R. precentral gyrus	-3.46	0.0007
L. superior frontal gyrus and R. postcentral gyrus	-3.46	0.001
L. inferior frontal gyrus and R. postcentral gyrus	-3.44	0.0009
R. inferior frontal gyrus and R. precentral gyrus	-3.4	0.0007
L. lateral orbitofrontal gyrus and R. inferior frontal gyrus	-3.38	0.0007
R. inferior frontal gyrus and R. superior temporal gyrus	-3.37	0.0008
L. lateral orbitofrontal gyrus and R. middle frontal gyrus	-3.32	0.0009
L. superior frontal gyrus and R. superior temporal gyrus	-3.29	0.001
L. middle frontal gyrus and R. inferior frontal gyrus	-3.26	0.0011
L. inferior temporal gyrus and R. postcentral gyrus	-3.2	0.0014
R. inferior frontal gyrus and R. postcentral gyrus	-3.2	0.0016
L. middle orbitofrontal gyrus and L. precentral gyrus	-3.17	0.0017
L. middle orbitofrontal gyrus and R. postcentral gyrus	-3.16	0.002
L. superior frontal gyrus and R. inferior frontal gyrus	-3.03	0.0024
L. inferior temporal gyrus and R. inferior frontal gyrus	-2.97	0.003
L. precentral gyrus and R. superior temporal gyrus	-2.97	0.0031
L. inferior frontal gyrus and R. superior temporal gyrus	-2.91	0.0046
L. inferior frontal gyrus and R. inferior frontal gyrus	-2.84	0.0047
L. lateral orbitofrontal gyrus and R. superior temporal gyrus	-2.83	0.0047
Regions with higher coherence in ASD		
R. angular gyrus and R. fusiform gyrus	4.38	<0.0001
L. angular gyrus and R. angular gyrus	4.13	<0.0001
L. superior occipital gyrus and R. angular gyrus	3.92	0.0001
R. angular gyrus and R. cuneus	3.91	0.0001
R. fusiform gyrus and R. supramarginal gyrus	3.87	0.0002
L. middle occipital gyrus and R. angular gyrus	3.8	0.0001
L. superior parietal gyrus and R. fusiform gyrus	3.71	0.0003
R. angular gyrus and R. inferior temporal gyrus	3.67	0.0002
L. lingual gyrus and R. angular gyrus	3.61	0.0003
R. fusiform gyrus and R. middle occipital gyrus	3.59	0.0004
R. angular gyrus and R. middle occipital gyrus	3.54	0.0004
R. angular gyrus and R. lingual gyrus	3.49	0.0006
L. middle occipital gyrus and R. middle occipital gyrus	3.44	0.0007
L. inferior occipital gyrus and R. angular gyrus	3.39	0.0007
L. angular gyrus and R. middle occipital gyrus	3.38	0.0007
L. superior occipital gyrus and R. supramarginal gyrus	3.36	0.0008
R. fusiform gyrus and R. gyrus rectus	3.34	0.0014
L. angular gyrus and L. superior temporal gyrus	3.32	0.0009
L. angular gyrus and R. fusiform gyrus	3.32	0.001
R. cuneus and R. supramarginal gyrus	3.29	0.001
R. angular gyrus and R. inferior occipital gyrus	3.28	0.001
L. angular gyrus and R. supramarginal gyrus	3.25	0.0014
L. superior occipital gyrus and R. superior frontal gyrus	3.14	0.0017
R. inferior temporal gyrus and R. middle occipital gyrus	3.14	0.0018
L. angular gyrus and R. inferior temporal gyrus	3.12	0.0018
R. angular gyrus and R. middle orbitofrontal gyrus	3.1	0.002
L. gyrus rectus and L. superior occipital gyrus	3.1	0.0031
L. angular gyrus and L. lateral orbitofrontal gyrus	3.06	0.0022
L. superior parietal gyrus and R. inferior temporal gyrus	3.04	0.0024
L. superior occipital gyrus and R. middle occipital gyrus	2.99	0.0032
R. angular gyrus and R. lateral orbitofrontal gyrus	2.98	0.0028
L. superior occipital gyrus and R. lateral orbitofrontal gyrus	2.98	0.003
L. middle occipital gyrus and R. fusiform gyrus	2.93	0.0034
R. gyrus rectus and R. inferior temporal gyrus	2.93	0.0042
L. angular gyrus and L. middle occipital gyrus	2.91	0.0037
L. inferior occipital gyrus and R. fusiform gyrus	2.9	0.0038
L. fusiform gyrus and R. angular gyrus	2.9	0.0046
L. angular gyrus and R. lateral orbitofrontal gyrus	2.85	0.0044

Positive *z*-scores reflect higher coherence in ASD. Negative *z*-scores reflect higher coherence in NT.

**P <0.01.

***P <0.001.

****P <0.0001.

**Figure 5:**
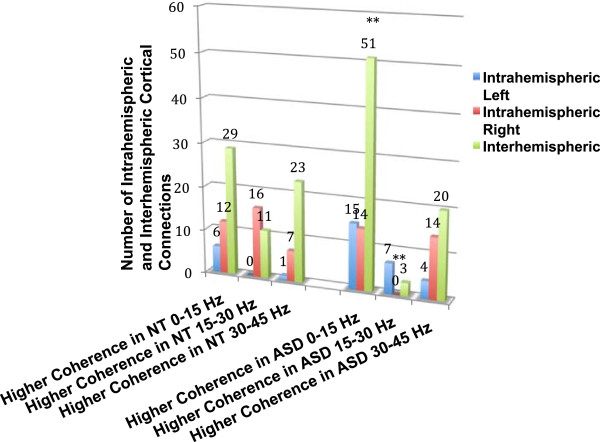
**Intra- and inter-hemsispheric cortical differences in synchronous beta and gamma activity during averted gaze.** Number of intra- and inter-hemispheric cortical differences within the low (0 to 15 Hz), beta (15 to 30 Hz), and low gamma (30 to 45 Hz) frequency bands in regions of either statistically higher coherence in NT or ASD participants.

Regarding intra- and inter-hemispheric differences in coherence, there was a significant association between group membership and the number of coherent right intra-hemispheric connections *X*^2^(2) = 8.34, *P* <0.01. Essentially no right intra-hemispheric coherent connections were noted in the 15 to 30 Hz range during passive viewing of averted gaze in ASD. No association between group membership and the number of coherent left intra-hemispheric connections was noted *X*^2^(2) = 2.57, *P* = 0.27 There was also a significant association between group membership and the number of inter-hemispheric coherent connections *X*^2^(2) = 10.01, *P* <0.007. More specifically, the ASD participants had a 6.5 times greater number of low frequency inter-hemispheric coherent connections relative to coherent beta and gamma frequency connections than NT while viewing averted gaze. See Figure 5 to examine differences in intra- and inter-hemispheric cortical connections within the low, beta, and low gamma frequency bands in regions of either higher coherence in NT or ASD participants during averted gaze.

### Relationship between oscillatory activity and clinical symptomatology

ADI-R scores were significantly correlated with coherence values in a predictable pattern among the ASD group under both the direct and averted gaze conditions (see Table 5). During direct gaze and collapsed over all frequencies, significantly higher coherence between left parietal regions (that is, angular and supramarginal gyri) was related to a greater number of ASD symptoms as reported on the ADI-R (≤0.005) while higher coherence between bilateral frontal cortical regions was related to fewer ASD symptoms. That is, between left inferior frontal/lateral orbitofrontal and right precentral gyri (≤0.01 and ≤0.005, respectively).

**Table 5:** Significant Kendall Tau correlation coefficients between the ADI-R and brain region pairs with statistically significant between group differences in coherence for averted gaze for each frequency band and direct gaze collapsed across frequency band

**Averted gaze**						**Direct gaze**	
**0 to 15 Hz**	**τ**	**15 to 30 Hz**	**τ**	**30 to 45 Hz**	**τ**	**Collapsed across all frequencies**	**τ**
L. angular gyrus and L. lingual gyrus	0.55***	L. lateral orbitofrontal gyrus and R. postcentral gyrus	-0.53**	L. angular gyrus and R. lateral orbitofrontal gyrus	-0.55***	L. angular gyrus and L. supramarginal gyrus	0.46***
L. angular gyrus and L. middle temporal gyrus	0.56***	L. middle frontal gyrus and R precentral gyrus	-0.60***	L. inferior frontal gyrus and R. precentral gyrus	-0.66****	L. inferior frontal gyrus and R. precentral gyrus	-0.41**
L. angular gyrus and L. superior temporal gyrus	0.60***	L superior occipital gyrus and R. inferior occipital gyrus	-0.52**	L. inferior occipital gyrus and R. fusiform gyrus	0.57***	L. lateral orbitofrontal gyrus and R. precentral gyrus	-0.44***
L. angular gyrus and R. angular gyrus	0.54***	R. inferior frontal gyrus and R. middle frontal gyrus	-0.51**	L. inferior temporal gyrus and R. precentral gyrus	-0.59***		
L. angular gyrus and R. middle temporal gyrus	0.50**	R. inferior frontal gyrus and R. superior frontal gyrus	-0.63****	L. lateral orbitofrontal gyrus and R. precentral gyrus	-0.57***		
L. inferior frontal gyrus and R. middle frontal gyrus	-0.56***	R. middle orbitofrontal gyrus and R. precentral gyrus	0.59***	L. lingual gyrus and R. angular gyrus	0.57***		
L. inferior temporal gyrus and R. cuneus	0.66****	R. superior frontal gyrus and R. superior temporal gyrus	-0.52**	L. middle frontal gyrus and R. precentral gyrus	-0.53**		
L. middle occipital gyrus and L. middle temporal gyrus	0.52**			L. middle occipital gyrus and R. fusiform gyrus	0.51**		
R. angular gyrus and R. superior frontal gyrus	-0.56***			L. precentral gyrus and R. superior temporal gyrus	-0.50**		
R cuneus and R. gyrus rectus	-0.59***			L. superior frontal gyrus and R. superior temporal gyrus	-0.50**		
R middle frontal gyrus and R superior frontal gyrus	-0.74****			L. superior occipital gyrus and R. lateral orbitofrontal gyrus	-0.60***		
				R. angular gyrus and R. cuneus	0.64****		
				R. angular gyrus and R. fusiform gyrus	0.62***		
				R. angular gyrus and R. inferior occipital gyrus	0.51**		
				R. angular gyrus and R. inferior temporal gyrus	0.53**		
				R. angular gyrus and R. lingual gyrus	0.64****		
				R. angular gyrus and R. middle occipital gyrus	0.71****		
				R. angular gyrus and R. middle orbitofrontal gyrus	0.56***		
				R. fusiform gyrus and R. middle occipital gyrus	0.61***		
				R. inferior temporal gyrus and R. middle occipital gyrus	0.53**		

**P ≤0.01.

***P ≤0.005.

****P ≤0.001.

A similar pattern of relationships was noted during the averted gaze condition in the low frequency band. Significantly higher coherence between unilateral and bilateral parietal regions (*P* ≤0.005), between left parietal and left occipital cortices (*P* ≤0.005) as well as between left parietal and bilateral temporal regions (*P* ≤0.005), and between left temporal and bilateral occipital regions (R *P* ≤0.005; L *P* ≤0.01) was related to a higher scores on the ADI-R and more severe ASD symptoms. In contrast, higher coherence between unilateral right frontal (*P* ≤0.001) and bilateral frontal regions (*P* ≤0.005) as well as between right frontal and right parietal (*P* ≤0.005) and right frontal and right occipital (*P* ≤0.005) was related to fewer ASD symptoms.

Primarily negative correlations were obtained during the averted gaze condition in the beta frequency band. Higher beta frequency band coherence between bilateral frontal regions (*P* ≤0.005), between unilateral right frontal regions (*P* ≤0.001 - right inferior and superior frontal gyri; *P* ≤0.01 - right inferior frontal and middle frontal), between right frontal and right temporal cortices (*P* ≤0.01), and unexpectedly, between bilateral occipital regions (*P* ≤0.01) was related to lower scores and fewer ASD symptoms in the ADI-R. Higher symptom reporting was only related to increased coherence between the right precentral and right orbitofrontal regions.

In the gamma frequency band, higher coherence between bilateral frontal regions (particularly right precentral gyri) and all other cortical regions bilaterally was related to a lower number of ASD symptoms as reported on the ADI-R. Higher gamma frequency band coherence between the right precentral gyrus and left inferior frontal (*P* ≤0.001), left lateral orbitofrontal (*P* ≤0.005), left middle frontal (*P* ≤0.01), and the left inferior temporal cortex (*P* ≤0.005) was related to lower scores and fewer symptoms on the ADI-R. Higher gamma band coherence between right lateral orbitofrontal and left superior occipital (*P* ≤0.005) and the left angular gyrus (*P* ≤0.005) was also related to fewer ASD symptoms. Finally, higher gamma band activity between the right temporal gyrus and left superior frontal gyrus (*P* ≤0.01) was related to lower scores on the ADI-R.

An opposite and strongly lateralized right hemispheric finding was also obtained when examining gamma activity. Higher gamma frequency band coherence between the right parietal cortex (that is, angular gyrus) and right orbitofrontal (*P* ≤0.005), temporal (*P* ≤0.005 - fusiform; *P* ≤0.01 - inferior temporal), and occipital regions (*P* ≤ .001 - lingual and cuneus; *P* ≤0.005 - inferior occipital) as well as between bilateral occipital cortical regions and right temporal cortex (*P* ≤0.005 - bilateral fusiform; *P* ≤0.01 - inferior temporal) was related to a greater number of ASD symptoms as reported on the ADI-R.

## Discussion

Processing eye gaze is a vital ability as it provides socially relevant information about one’s environment, allows us to make inferences about the possible intentions of others, and is one of the most important aspects of non-verbal communication. Although gaze processing deficits are a seminal, early, and enduring behavioral deficit in ASD, a comprehensive characterization of the neural processes mediating abnormal gaze processing in ASD has yet to be conducted.

MEG studies exploring other well-described behavioral phenomena, such as deficits in face and emotion processing [47-50] , have recently been reported following the growth of more unified theories that include components of altered connectivity, an imbalance in excitatory to inhibitory neural transmission, and impaired neural synchrony as fundamental pathophysiological mechanisms of ASD. These preliminary studies have reported findings suggestive of abnormal functional organization, aberrant pathway development, and possible altered hemispheric specialization.

Recent investigations have also explored neural synchronization (phase coherence) and connectivity during both face [33,51] and auditory/language processing in ASD in an attempt to better elucidate aberrant patterns of connectivity and identify potential biomarkers [52-56], with a specific focus on gamma power and oscillatory activity. The results are generally equivocal with studies reporting higher regionally induced gamma power, higher and lower regionally evoked gamma power, and reductions in phase consistency or timing of power (that is, phase locking). This may be partially accounted for by the rather significant heterogeneity in this population coupled with the extreme variability in the methodologies being used and populations being examined.

Resting state investigations exploring oscillatory activity have only very recently emerged [57-59] consistent with efforts to more fully characterize global aberrant connectivity patterns in ASD. Indeed, Tsiaras *et al.*[59], reported finding attenuated short-range connectivity in adults with ASD within bilateral temporal and frontal regions and left parietal regions, although significant differences between specific frequency bands were not apparent. In a study of resting state in children with ASD compared to typically developing children, Cornew and colleagues [57] recently reported finding various differences in oscillatory activity including increased theta and alpha power in parietal and occipital regions and additionally increased alpha power in temporal regions in ASD. The authors further reported finding greater relative delta in right frontal regions in ASD. These results are generally consistent with neurophysiological findings from EEG [60], but they fail to bring us closer to a comprehensive understanding the relationship between the behavioral phenotype and the pathophysiological mechanisms of this disorder.

**Figure 6:**
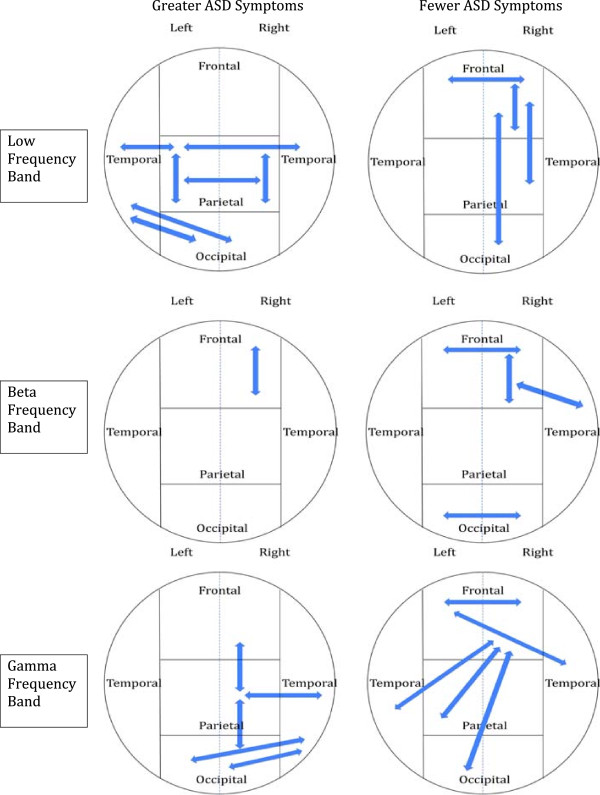
**Relationships between oscillatory activity in the low, beta, and low gamma frequency bands during averted gaze and autism symptomatology measured by the ADI-R.** Note increased low and gamma frequency band oscillatory connections in posterior temporo-parieto-occipital regions associated with a higher number of ASD symptoms while high frequency gamma oscillatory connections between right frontal regions and all left hemisphere lobes is associated with fewer ASD symptoms.

Given its central phenotypic prominence [19-23] in ASD, gaze processing clearly surfaces as a strong and enduring endophenotypic candidate. In order to more broadly understand the relationship between a core and enduring behavioral deficit in ASD and its neuropathology, we hypothesized *a priori* that processing eye gaze information was likely to more precisely characterize aberrant beta and gamma band oscillatory activity and potentially aberrant connectivity. Our results are very consistent with the aforementioned investigations and revealed that ASD participants demonstrated lower coherence between bilateral frontal (particularly right frontal) and right pre-and postcentral regions and superior temporal regions when passively viewing gaze. In contrast, ASD participants demonstrated higher coherence between sensory association cortices (that is, temporo-parieto-occipital) in all frequency bands, particularly within the low frequency range, as well as in those associated with both short- and long-range transmission.Our results also reveal a very clear relationship between aberrant oscillatory activity and elevated ASD symptoms, specifically an increase in low frequency activity posteriorally and bilaterally as well as an increase in gamma activity in right posterior temporo-parietal-occipital regions. In contrast, lower symptomatology appears related to increased low frequency coherent activity between the right frontal cortex with left frontal and right parietal and occipital regions as well as related to increased oscillatory activity in the gamma frequency band between right frontal regions with all other left hemisphere lobes (frontal, temporal, parietal, and occipital) and between the left frontal and right temporal lobes (see Figure 6). The regional brain differences noted in ASD participants displaying fewer clinical features are very consistent with the purported brain regions known to underlie social cognition.

### Disruptions in neuroanatomical pathways

Recent multi-modal imaging methods have started to illuminate networks involved in direct and averted gaze in NT using, for example, combined fMRI-DTI [61]. Dynamic gaze shifts have been found to increase activation in a well-established region of the social network including the right pSTS, anterior insula, and fusiform gyrus with direct connections noted between the right pSTS and anterior insula. These regions are thought to be supported by long-range network connections that project via the superior longitudinal fasciculus and are believed to be critical for extracting social meaning of eye gaze shifts [61]. Consistent with this purported social network, compared to those with ASD, our NT participants demonstrated significantly higher coherence between the right middle and inferior frontal gyri and the right superior temporal regions within the beta band frequency known to be critical for long-range connectivity.

In contrast, our ASD participants demonstrated higher gamma power in inter-hemispheric connections between the left and right parietal lobes and intra-hemispherically between the right parietal lobe (angular gyrus) and temporal regions. The angular gyrus has been implicated in a number of processes including reading and number comprehension, numerical processing, visual attention, and social cognition. It is a cross-modal region where sensory information from the visual, auditory, and tactile senses converge allowing for a combined and integrated percept. It is essential for the manipulation of mental representations and reorienting of attention [62]. We believe that heightened connectivity in these regions without appropriate regulatory or contextual feedback from frontal regions may result in exquisite sensory sensitivity, acceleration of letter, number, and word recognition with limited comprehension or applied skills, or an over-allocation of attention to information without a clear appreciation of its relevance; a neurocognitive pattern often noted in ASD. A strengthening of connectivity between the right angular gyrus and inferior temporal regions without frontal mediation, particularly from medial prefrontal regions, may further contribute to a heightened attention to the invariant features of the face or its components without an ability to extract essential social relevance. Our findings are consistent with models of ASD proposed by Brock and colleagues [25] as well as Belmonte *et al.*[27] who have suggested that abnormally high levels of high-frequency neural activity and over connectivity within localized brain regions causes impaired discrimination of brain signal from background brain noise. Interestingly and unexpectedly, our ASD participants demonstrated a greater number of low frequency cross-hemispheric connections, and particularly in posterior cortical regions. The results suggest that conceptualizing ASD as a disorder of either over- or under-connectivity in long- or short-range connections may not fully capture the pathophysiology.

### Synchronous oscillatory activity during gaze cueing as a biomarker of ASD?

Approximately 200 investigations have been published proposing neuroanatomical markers of ASD, although the results have often been in conflict or unreplicated [63]. The majority of this work has been in older children and adults using structural imaging. Recently, Ecker and colleagues [64] used linear support vector machines (SVM), a machine learning method that identifies patterns in data by identifying hyperplanes that maximally differentiate categories or groups, classified 20 adults with and without ASD. They used five structural classifiers of cortical gray matter to correctly classify 85% of those with ASD, with 90% sensitivity and 85% specificity. Left hemispheric classifiers were more accurate than right, and cortical thickness was the best classifier. Hemisphere laterality is an area that remains relatively unexplored in ASD, and with their reported method the authors were unable to determine if individuals with ASD displayed a higher (lower) degree of cortical asymmetry. Similarly, a recent fMRI investigation by Dinstein and colleagues [29] of sleeping toddlers with ASD revealed significantly weaker inter-hemispheric synchronization (that is, weak ‘functional connectivity’ across the two hemispheres) in inferior frontal (IFG) and superior temporal (STG) regions for which early ‘over-lateralization’ of language function was suggested. However, again, directionality of lateralization to the left or right hemisphere could not be determined from their data. Our method allows us to provide a direct numerical comparison between pathways, both inter- and intra-hemispherically, and to examine group differences between frequency bands known to underlie short- and long-range connectivity. Given the passive nature of the task, this methodology could be easily applied to preschoolers. Only 18% of children with ASD are identified by the age of 3 years; even later for children with milder forms (average, 6.3 years) [65]; well beyond when children can benefit maximally from early intervention. This method allowed coherence to be imaged in source rather than sensor space, achieving a significant degree of disentanglement of signals. Moreover, it provided better resolution and precision of the underlying networks generating the signals since coherence in sensor space is smeared due to current spread as described by Srinivasan *et al.*[66]. Neural synchrony between the frontal cortex and pre- and postcentral gyrus, between frontal and superior temporal cortex in the beta band, and between angular and fusiform gyri in the gamma band were particularly discrepant between the groups.

We recognize that there are a number of weaknesses with this pilot study. First, the sample size of this pilot phase was small which limits the external validity and conclusions that can be drawn. Although the findings were quite robust, a replication of this study with a larger number of participants is necessary. Moreover, the results suggest a strong need for funding of large scale and longitudinal studies with this technology in ASD.

Second, our ASD participants remained on their psychotropic medication regimens during the study given the perceived cost-benefit of motion artifact for participants taking ADHD medications as well as the practical challenges associated with withdrawing from medications with longer half-lives. However, we recognize that when one is evaluating a possible pathophysiological mechanism in any psychiatric or neurodevelopmental disorder, a vital issue is whether the observed neural abnormality is present at the outset of the disorder and predates medication exposure and the deleterious effects of long-term illness. As noted, five of our participants were on one or more of the following medications: psychostimulants, antidpressants, anxiolytics, antipsychotics, or mood stabilizers. Recent investigations exploring alterations in oscillatory activity in populations taking medications similar to our cohort (for example, ADHD, bipolar disorder, and schizophrenia) have reported variable results with respect to medication status on oscillatory activity and the findings to date have been primarily reported in adult populations [67-69]. Wilson and colleagues [69] recently examined time estimation and oscillatory activity using MEG in medicated and unmedicated individuals with ADHD. Relative to controls, unmedicated participants were reported to display less accurate time estimation and weaker gamma activity in frontal cortex, specifically anterior cingulate, supplemental motor areas, and the left prefrontal cortex. Following medication administration, the patients demonstrated small but significant increases in gamma-band activity across the same neural regions, which was related to improved time estimation accuracy. Exploratory analysis also revealed stronger delta activity in frontal regions in the control participants relative to those with ADHD, regardless of medication status, possibly suggesting that such alterations in delta activity may not be responsive to stimulant medications. Although we recognize it is difficult to compare across populations, recent investigations have demonstrated high co-morbidity with ADHD and ASD, suggesting similar aberrant neural substrates. We further reviewed the literature to explore possible effects of antipsychotics on oscillatory power. Minzenberg and colleagues [67] recently reported reductions in gamma oscillatory power during an executive task that was independent of medication status in first-episode schizophrenia. Fifty-three first episode patients with schizophrenia (21 without antipsychotic treatment), aged 13 to 30 years, underwent EEG while performing a cognitive control task. Both the medicated and unmedicated patient subgroups were impaired on the behavioral task and displayed lower frontal induced gamma power. In contrast, there were no significant group differences in theta power between controls or the medicated or unmedicated patient subgroups. Given the above, we might have expected the effect of psychostimulants and antipsychotics to attenuate the frontal gamma frequency band group differences noted between our groups. Özerdem *et al.*[68] examined alpha and beta oscillatory activity during a visual odd-ball paradigm and EEG in 10 bipolar patients before and after valproate treatment. At baseline, drug-free individuals with bipolar disorder in the hypomanic or manic phase of illness demonstrated aberrant alpha and beta oscillatory activity to visual target stimuli compared to healthy controls. Individuals with bipolar disorder displayed significantly higher beta activity in occipital cortices compared to controls, and patients were devoid of an occipital-frontal alpha dominance that was noted in the control group. Following treatment with valproate, occipital beta responses dropped to control levels, and a further and significant decrease in occipital alpha and unchanged frontal alpha response was noted in patients with bipolar disorder. The authors suggest that the latter may suggest the inhibitory effect of valproate via GABAergic inhibition. That is, inhibition of occipital alpha activity while trying to manage cortical hyperactivity may be an unwanted effect of valproate use. Similarly, if the effect of valproate is an attenuation of occipital alpha activity, the impact of this medication would have also been to decrease our current effect of increased low frequency band activity in posterior regions. However, these assumptions should be interpreted cautiously as it is important to remember that investigations examining auditory and visual evoked oscillatory activity in humans have shown that the evoked responses are topography and stimulus modality dependent and that brain structures with different resonance properties may be dependent on the stimuli.

Finally, with respect to the frequency ranges that were examined, while we did not examine activity greater than 45 Hz, we recognize that the gamma band frequency extends to approximately 100 Hz. For this pilot project, we chose to remain below 60 Hz given that group differences in gamma power are the most robust in the 40 Hz range and in order to avoid additional artifact. Despite these limitations, this pilot study provided the first cogent understanding of whole-brain patterns of coherence that underlie direct and averted eye gaze in ASD.

## Conclusions

One of the hallmarks of ASD is a failure to detect and/or respond in a typical manner to information conveyed by eye gaze. This study characterized whole-brain patterns of synchrony in ASD compared to NT during direct and averted eye gaze processing while undergoing MEG. Results revealed: (1) higher coherence and synchronization in temporo-parietal-occipital brain regions across all frequencies in ASD, particularly within the low frequency range; (2) a higher number of low frequency cross-hemispheric coherent connections; and (3) a near absence of right intra-hemispheric synchrony in the beta frequency band in ASD. This preliminary examination of the relationship between neural synchrony and ASD symptomatology further revealed a unique pattern of relatively mutually exclusive findings: (1) higher synchronization in the low frequency band (0 to 15 Hz) between left temporo-parieto-occipital regions and higher synchronization in the low gamma frequency band (30 to 45 Hz) in right temporo-parieto-occipital regions was associated with more severe ASD symptomatology while (2) higher synchronization in the low frequency band between right front-parieto-occiptal regions as well as in the gamma frequency band between bilateral frontal and right frontal with left temporo-parieto-occipito regions was associated with fewer ASD symptoms. This altered pattern of oscillatory activity may contribute to aberrant connectivity that underlies the failure of individuals with ASD to appropriately orient to eye gaze, which has a cascading negative effect on typical social and language development.

## Consents

Informed consent and/or assent were obtained on all participants of the study following guidelines established by the Institutional Review Boards of the participating institutions.

## Competing interests

The authors declare that they have no competing interests.

## Authors’ contributions

RLO and SMB conceived of the study and its design, were responsible for study coordination, assisted with data collection and analysis, and drafted the manuscript. RLO, JM, AR, and SMB were responsible for the design of the MEG methodology, data acquisition, and data analysis. AO, LP, DJ, AM, KV, LAE, and AMM were responsible for subject recruitment, psychometric and diagnostic assessments, and MEG data analysis. All authors read and approved the final manuscript.
